# Performance of granular ferric hydroxide process for removal of humic acid substances from aqueous solution based on experimental design and response surface methodology

**DOI:** 10.1016/j.mex.2018.12.010

**Published:** 2018-12-18

**Authors:** Mahmood Yousefi, Ramin Nabizadeh, Mahmood Alimohammadi, Ali Akbar Mohammadi, Amir Hossein Mahvi

**Affiliations:** a Department of Environmental Health Engineering, School of Public Health, Tehran University of Medical Sciences, Tehran, Iran; b Center for Solid Waste Research, Institute for Environmental Research, Tehran University of Medical Sciences, Tehran, Iran; c Department of Environmental Health Engineering, Neyshabur University of Medical Sciences, Neyshabur, Iran

**Keywords:** Adsorption, Humic acid, Aqueous solution, Granular ferric hydroxide, RSM

## Abstract

Response surface methodology has been used to design experiments and to optimize the effect of independent variables responsible for higher adsorption of humic acid (HA) by granular ferric hydroxide (GFH) from aqueous solutions. The variables of pH (3–11), contact time (15–120 min), adsorbent dose (1–5 g/L) and initial concentration of humic acid (5–20 mg/L) were examined. The adsorption isotherms and kinetics of humic acid substances on granular ferric hydroxide (GFH) were studied. Also the design of the experiments was performed using R software by the CCD (central composite design) method. Variance analysis (ANOVA) was used as the statistical response analysis method. Result of this study proved the optimal values of the independent variables of the adsorbent dose, contact time, initial concentration of humic acid and pH were 4 g/L, 93.75 min, 16.25 mg/L, and 5, respectively. The experimental data followed the Langmuir isotherm and pseudo-second kinetic model. Based on the response surface methodology, higher HA removal efficiencies were obtained with acidic condition, longer reaction time, and appropriated loading amount of GFH.

## Graphical abstract

**Figure:**
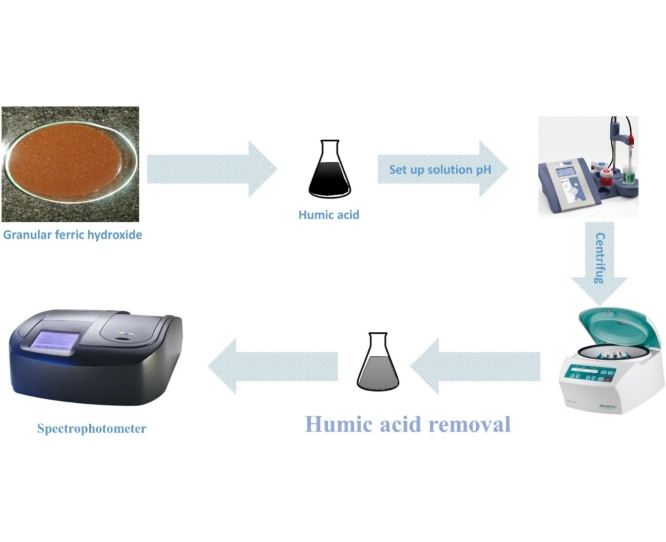

**Specifications Table**

**Table:** 

Subject area	Environmental science
More specific subject area	Adsorption
Protocol name	Application of granular ferric hydroxide adsorbent in the removal of humic acid from aqueous solutions.
Reagents/tools	Measurement of humic acid concentration was carried out using the DR 5000 spectrophotometer) HACH model(with UV/vis detector at the wavelength of 254 nm. A digital pH meter (Basic 20 Crison) was used for solution pH analyzing.
Experimental design	Determining the humic acid concentrations under various levels of initial humic acid concentration, solution pH, and reaction time to obtain optimal removal of humic acid from aqueous solution using a granular ferric hydroxide
Trial registration	No applicable
Ethics	No applicable

## Protocol data

• The result of this study show that the adsorption capacity of granular ferric hydroxide is high and hence, is a capable adsorbent for removing humic substance (HS) from aqueous solution.
• The result of response surface model shows that the experiments are highly accurate and the model is significant.
• Statistical analysis of data shows that small variations in the values of the selected variables alter the HA removal efficiency.
• It can be concluded that granular ferric hydroxide (GFH) have desirable quality, cost-effective, and high efficiency to the problem of humic substance (HS) from surface water

## Description of protocol

### Preparation of granular ferric hydroxide

**Fig. 1:**
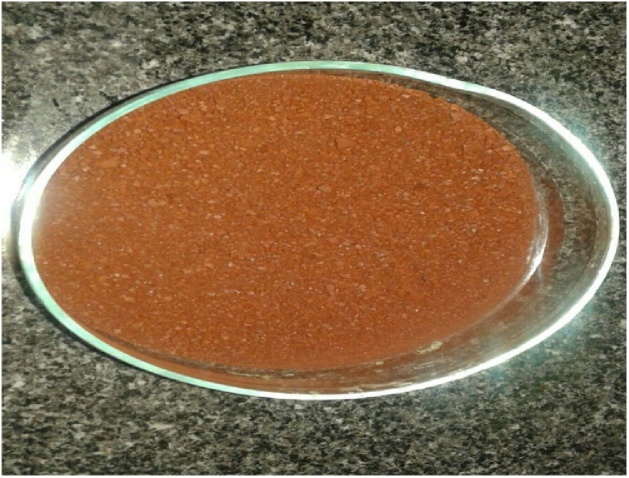
Granular ferric hydroxide used in the study.

Granular ferric hydroxide (GFH) is an adsorbent, developed at the environmental engineering especially for selective removal of natural organic matter from aqueous solution. Fig. 1 shows the absorbent used in the study. The characteristics of GFH used in this study is reported in Table1. In order to removal of the moisture, GFH was dehydrated in the oven (105 °C) for 90 min and also, has been place in the desiccator for cooling [[3], [4], [5]].

**Table 1:** GFH properties used in experiments.

Property	Unit	Value
Saturation	%	43–48
Porosity	%	72–77
pH	na	7.5–8.2
Specific surface	m^2^/m^3^	280
Effective size	mm	0.32–1
Uniformity coefficient	na	About 3

### Adsorption process studies

After preparation of the GFH, required solutions of a certain concentration were also prepared. Distilled water was used for preparation of all these solutions. The experimentations were carried out in the Chemistry laboratory of Environmental Health Engineering Department in Tehran University of Medical Sciences. Then, the factors affecting the performance of the studied processes, like the initial concentration of the humic acid, pH contact time and adsorbent dose were investigated. Isotherm and kinetics were also studied.

For working in a discontinuous system, a 250 ml Erlenmeyer flask was used.

In each step, a certain volume of humic acid solution and a specific dose of GFH were added to Erlenmeyer flask. Also the desired conditions in experiment were adjusted. In the following, mixing process (250 rpm) was carried out by use of a shaker and after passing through the filter, considering the required concentrations, the desired volume of the storage solution was removed and the humic acid was analyzed. The humic acid of removal efficiency was determined by adsorbent of granular ferric hydroxide using the following equation:

(1) $$\%\text{RE}=\frac{\left({\text{C}}_{\text{i}}-{\text{C}}_{\text{t}}\right)}{{\text{C}}_{\text{i}}}\times 100$$

Where, C_i_ and C_t_ are the initial concentrations and concentrations at time t, respectively.

### Preparation humic acid solutions and measurement of humic acid concentration

In order to running the adsorption experiment, in the first step, the required solutions of humic acid was prepared. The humic acid was purchased from Acros Co. which had some impurities and in order to eliminate this impurity, 1.82 g of humic acid with a purity of 57% was weighed. Purification was performed by the method of Zakoni et al., as follows: after filtration, the humic acid was mixed with potassium hydroxide 0.2 M and potassium chloride 0.3 M for 4–5 h. Afterwards, centrifugation was done to remove dissolved material such as humic acid. In order to coagulate the humic acid and centrifuging, the floated liquid reached pH = 0.5 by the Hydrochloric acid. Humic acid, which is relatively pure, was washed again with deionized water and Hydrochloric acid and re-centrifuged. Then, deionized water was added to deposited materials and they reached a volume of 1 L in a flask. The solution was then poured into a beaker and agitated for three hours. The solution obtained after three hours of agitation was purified humic acid with concentration 1 g/L, which was stored at 4 °C [1,2,[6], [7], [8], [9], [10]].

Measurement of humic acid concentration was carried out by the DR 5000 spectrophotometer of HACH model with UV/vis detector at the wavelength of 254 nm [1,2].

### Experimental design

The central composite design was used to determine the interaction of parameters pH, contact time, adsorbent dose and initial concentration of humic acid on the removal process and optimization of the removal process. Also, in the different stages of determining the number of empirical tests, the RSM was used for modeling and optimization. Data analysis was performed using the R software, version 3-3-2 (2016-10-31) and Microsoft Excel version 2016 for plotting calibration curves and basic mathematical calculations. The Solver plugin in Microsoft Excel software was used to determine the optimal values ​​for the study variables. Table 2 shows the experimental ranges used in central composite design (CCD) design for humic acid adsorption in the study. For humic acid, our preliminary guess in order to optimize the same central points of 16.25, 4, 93.75 and 5 was respectively, for the concentration of humic caid, the adsorbent dose, contact time and pH.

**Table 2:** Experimental ranges used in CCD design for humic acid adsorption.

Parameter	Limiting range		Min	Max	Ave	Range
Humic acid concentration (X_1_)	−1	1	5	20	12.5	7.5
pH (X_2_)	−1	1	3	11	7	4
Contact time (X_3_)	−1	1	15	120	67.5	52.5
Adsorbent (X_4_)	−1	1	1	5	3	2

**Fig. 2:**
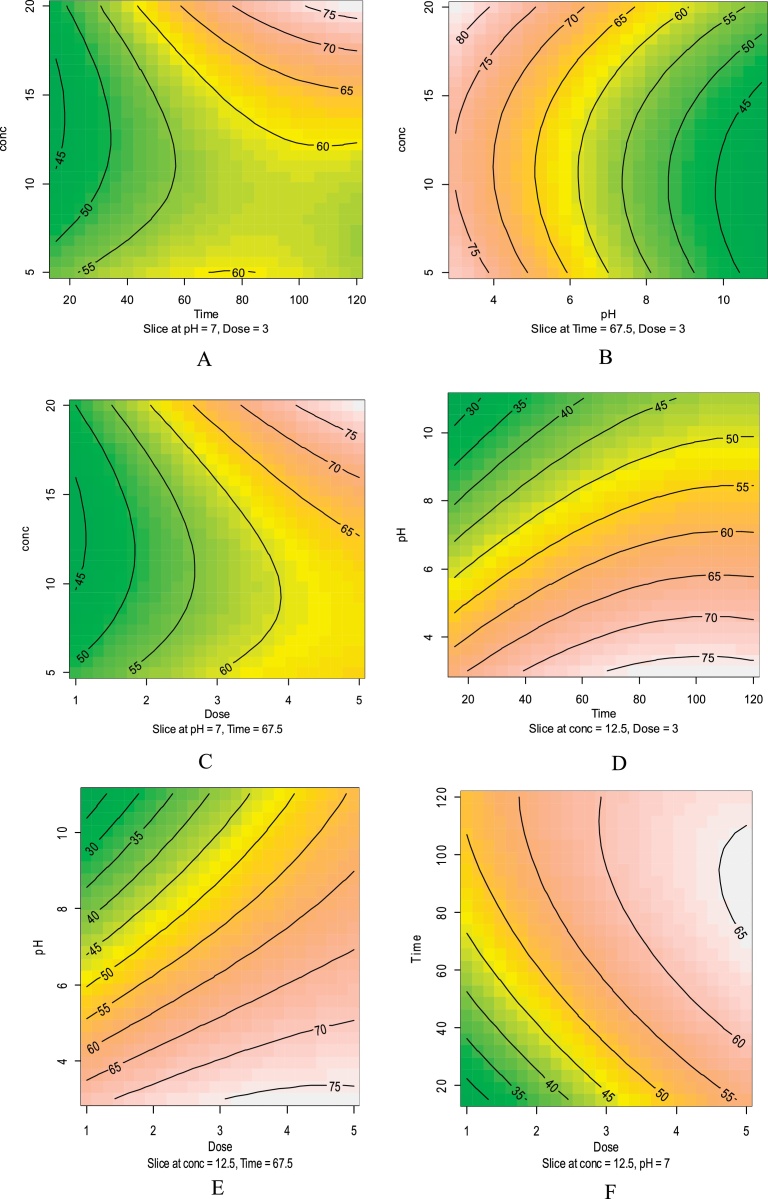
Contour plots for the interaction effect of variables on the humic acid removal. (A) humic acid concentration and pH, (B) humic acid concentration (mg/L) and contact time time(min), (C) pH and contact time time(min), (D) humic acid concentration (mg/L) and adsorbent dose (g/L), (E) pH and adsorbent dose (g/L), (F) contact time(min) and adsorbent dose (g/L).

**Fig. 3:**
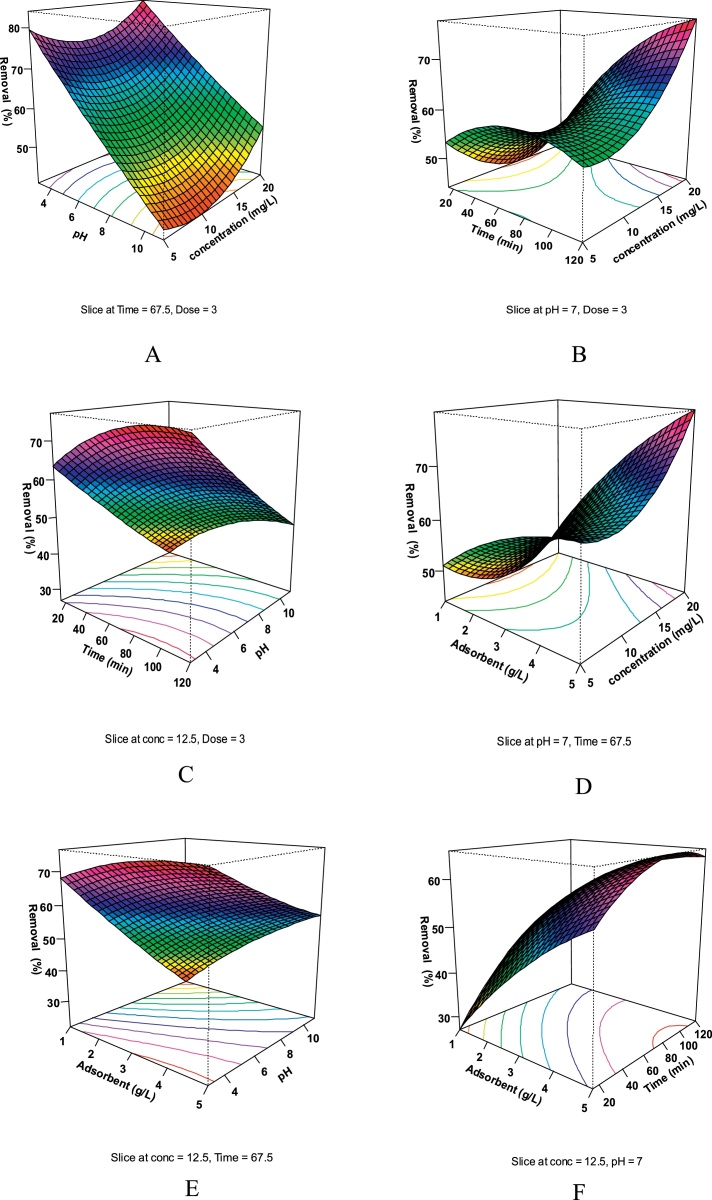
3D plots for the interaction effect of variables on the humic acid removal. (A) humic acid concentration and pH, (B) humic acid concentration (mg/L) and contact time (min), (C) pH and contact time (min), (D) humic acid concentration (mg/L) and adsorbent dose (g/L), (E) pH and adsorbent dose (g/L), (F) contact time (min) and adsorbent dose (g/L).

Contour and 3D plots for the interaction effect of variables on the humic acid removal are shown in Fig. 2, Fig. 3 also, regression analysis and analysis of variance (ANOVA) on the humic acid removal are shown in Table 3, Table 4.

**Table 3:** Regression analysis for humic acid removal.

Term of Model	Coefficient Estimate	Std. Error	t-value	Pr (>|t|)	p-value
Intercept	57.09091	0.48582	117.5144	<2.2e-16	***
X1	3.80833	0.6578	5.7895	1.15E-02	***
X2	−16.72500	0.6578	−25.4255	<2.2e-16	***
X3	8.07500	0.6578	12.2757	9.09E-08	***
X4	10.48333	0.6578	15.9369	7.83E-10	***
X1.X2	1.62500	1.61128	1.0085	3.25E-01	
X1.X3	6.37500	1.61128	3.9565	7.79E-04	***
X1.X4	4.37500	1.61128	2.7152	1.33E-02	*
X2.X3	1.72500	1.61128	1.0706	2.97E-01	
X2.X4	6.52500	1.61128	4.0496	6.27E-04	***
X3.X4	−3.82500	1.61128	−2.3739	2.77E-02	*
X1^2^	6.69242	1.14795	5.829	1.05E-02	***
X2^2^	1.04242	1.14795	0.9081	3.75E-01	
X3^2^	−4.90758	1.14795	−4.275	4.28E-01	***
X4^2^	−2.78258	1.14795	−2.424	0.0249558	*

Code of significance: 0 ‘***’ 0.001 ‘**’ 0.01 ‘*’ 0.05 ‘.’ 0.1 ‘’ 1.

**Table 4:** Analysis of variance (ANOVA) for humic acid removal.

Model formula in RSM	DF	Sum of squares	Mean square	F-value	Probability (>F)
First-order response	4	2816.01	704	271.16	<2.2e-16
Two-way interaction	6	122.6	20.43	7.87	0.0001911
Pure quadratic response	4	155.4	38.85	14.96	8.25E-05
Residuals	20	51.92	2.6	–	–
Lack of fit	10	25.84	2.58	0.9903	0.5060062
Pure error	10	26.09	2.61	–	–

### Determination of isotherms

The tests required to determine the isotherms of adsorption were performed after choosing the basic conditions of 4 g/L of adsorbent, the initial concentration of humic acid 5–30 mg/L at pH = 5. The linear form of the Langmuir equation used to investigate the adsorption phenomena is as follows [6,11,12,10]:

(2) $$\frac{C_{e}}{q_{e}}=\frac{1}{q_{m}}KL+\frac{C_{e}}{q_{m}}$$

Where qm is the maximum amount of humic acid where qm is the maximum of humic acid per a layer of humic acid in mg/g, KL is the constant of the absorption energy (l/mg) [[13], [14], [15]].

Freundlich equation does not predict maximum adsorption and the linear form of the equation is as follows. By illustration of the logarithmic curve qe as a function of the ce logarithm, the n, KF values can be calculated, which are constants of the Freundlich equation. K represents the amount of adsorption of humic acid per unit of equilibrium concentration and n indicates the distribution of particles of adsorbed materials which are bound to the surface of the adsorbent. Various values of 1/n between 0 and 1 denote the surface heterogeneity, and as n approaches zero, the surface heterogeneity increases [[8], [9], [10],^6^,^11^,^12^]. Using the following equation:

(3) $$\log\frac{x}{m}=\frac{1}{n}\log C_{e}+\log KF$$

Isotherm and their parameters for humic acid adsorption onto the GFH are shown in Table 5.

**Table 5:** Isotherm and their parameters for humic acid adsorption onto the GFH.

Isotherm	Parameters Values	
Langmuir	K_L_	1.204
	q_m_ (mg/g)	6.473
	R^2^	0.973
	R_L_	0.024225–0.129646
Freundlich	K_F_	3.084
	n	2.678
	R^2^	0.990

### Reaction kinetics

The adsorption process in kinetic studies is linear. The chemical reaction rate is expressed by the chemical kinetics. Most kinetic models for adsorption are of zero, 1 and 2 orders. Table 6 was shown the equations for adsorption kinetics, in which, k is the velocity coefficient and qe, qt are the adsorption capacity at equilibrium and at time t [[1], [2], [3], [4], [5]]. Kinetic and their parameters for humic acid adsorption onto the GFH are shown in Table 7.

**Table 6:** Kinetic equations.

Pseudo-first-order kinetic	$\ln\left(1-\frac{q_{t}}{q_{e}}\right)=k_{1}t$
Pseudo-second-order kinetic	$\frac{t}{q_{t}}=\frac{1}{k_{2}q_{e}^{2}}+\frac{1}{q_{e}}t$

**Table 7:** Kinetic model and their parameters for humic acid adsorption onto the GFH.

Kinetic model	Parameters	
Pseudo-first-order	K_1_	−0.09
	q_e_ (mg/g)	0.49
	R^2^	0.928
Pseudo-second-order	K_2_	0.39
	q_e_	1.08
	R^2^	0.997

## Conflict of interest

The authors of this article declare that they have no conflict of interests.
